# Rapid detection of avipoxvirus using a fluorescent probe-based multienzyme isothermal amplification assay

**DOI:** 10.3389/fvets.2025.1601685

**Published:** 2025-05-14

**Authors:** Yinchu Zhu, Suxin Huo, Liu Chen, Yuan Fu, Jionggang Hua, Tao Yun, Cun Zhang, Zheng Ni, Weicheng Ye

**Affiliations:** State Key Laboratory for Quality and Safety of Agro – Products, Institute of Animal Husbandry and Veterinary Science, Zhejiang Academy of Agricultural Sciences, Hangzhou, China

**Keywords:** avipoxvirus, multienzyme isothermal amplification, nucleic acid detection, rapid assay, clinic diagnostic

## Abstract

Avipoxvirus (APV) is a prevalent DNA virus in avian species, causing clinical symptoms of fowlpox and leading to reduced egg production, slower broiler growth, and increased mortality. The spread of APV poses a significant threat to the global poultry industry, potentially causing substantial economic losses. Effective control of APV, particularly its major species such as fowlpoxvirus and pigeonpoxvirus, requires the development of rapid and specific diagnostic tools. In this study, a novel multi-enzyme isothermal rapid amplification (MIRA) assay was developed to detect APV. Various primer-probe combinations were screened to identify an optimal pair targeting a conserved region of the viral *P4b* gene. The MIRA assay operates at a constant temperature and results can be visualized through fluorescence signal detection. The sensitivity, specificity, and applicability of the MIRA assay were evaluated. Additionally, 86 clinical samples were tested to assess the accuracy of the MIRA assay. The MIRA assay provides results within 15 minutes demonstrated high specificity, with no cross-reactivity with other avian pathogens. It achieved a detection limit of 50 copies/μl, which is consistent with the qPCR assay. Further evaluation with 86 clinical samples showed that the accuracy of the MIRA assay was comparable to that of qPCR in detecting fowlpoxvirus and pigeonpoxvirus. The results highlight the convenience, sensitivity, and rapidity of the MIRA assay as a promising tool for diagnosing APV.

## Introduction

Avipoxvirus (APV) is double-stranded DNA viruses with an enveloped structure and a genome size ranging from 260 kb to 360 kb, making them the largest and most structurally complex DNA viruses among animal (1). Avipoxviruses were first identified in 1873 and belong to the Chordopoxvirinae subfamily of the Poxviridae family (2). These viruses have been documented in over 300 species of birds, including both domesticated and wild birds (3). Avipox infections are typically spread through direct contact, and although transmission is generally slow, they can lead to significant economic losses in poultry industries due to reduced egg production, impaired broiler growth, blindness, and increased mortality (4). Wild bird populations are also severely affected by avian pox. APV includes approximately 12 known species, each associated with specific host species, such as Fowlpox virus (FPV), Turkeypox virus, Pigeonpox virus, Canarypox virus, Parrotpox virus, Flamingopox virus, Juncopox virus, Mynahpox virus, Penguinpox virus, Psittacinepox virus, Quailpox virus, and Sparrowpox virus (5).

According to the phylogenetics relationships of APVs’ 4b core protein coding gene demonstrated that there is divergence among the various virus were reliably connected with the varied hosts (6). Thus, the P4b conserved gene sequences were used to classify avipoxvirus (2). Therefore APVs were classified into 3 clusters, which were categorized as A fowlpox virus like, B canarypox virus like, C psittacine pox virus (7). The most common APV are pigeonpox virus (PPV), fowl-poxvirus (FPV).

The main approach to preventing avipox virus infections is vaccination. For example, FPV vaccines typically use live attenuated strains of FPV or other antigenically related APV strains, such as pigeonpox virus (8). The development of commercial vaccines by diverse veterinary pharmaceutical companies have played a crucial role in controlling avipox virus outbreaks. Currently, avipox viruses have been utilized as the vectors for the development of recombinant vaccines to prevent infection with homologous or heterologous pathogens (9).

Despite vaccination efforts, reports of FPV and PPV continue to emerge in poultry farms. Moreover, recent studies have shown that many field strains of FPV carry genome segments of the avian retrovirus, reticuloendotheliosis virus (REV), integrated into their genomes, which could potentially impair vaccine efficacy (10, 11). The role of backyard chickens and pigeons in spreading zoonotic viral diseases, which can have fatal consequences, as well as other viral diseases threatening the commercial poultry industry, requires further investigation. In this context, rapid diagnostic methods are essential and play a critical role during outbreaks. Conventional laboratory diagnostics for APV including histopathological examination, virus isolation by propagation on the chicken embryo chorioallantoic membrane (CAM) (12), cell culture and serological methods. Molecular identification of APV via polymerase chain reaction (PCR) is a more sensitive and rapid approach than traditional methods (13). Real-time PCR offers even greater accuracy in identifying APV from various samples (14). However, the widespread use of these techniques is limited by the need for skilled technicians, expensive equipment, and time-consuming processes. This highlights the urgent need for a rapid, reliable, and simple diagnostic assay, such as an isothermal amplification-based method, to address these challenges.

Isothermal nucleic acid amplification technology has rapidly advanced in recent years, offering the advantage of operating at a constant temperature and delivering results in approximately 20 min (15). This makes it a convenient and rapid tool for pathogen detection. Various isothermal amplification-based techniques have been established, including loop-mediated isothermal amplification (LAMP) (16, 17), recombinase polymerase amplification (RPA) (18, 19), and multi-enzyme isothermal rapid amplification (MIRA) (20, 21), all of which function at a constant temperature. MIRA, in particular, is a novel and fast isothermal technique for nucleic acid detection. The amplification products can be confirmed through gel electrophoresis, lateral flow dipstick (LFD), or fluorescence signal analysis (22, 23).

The present study aims to develop a novel MIRA based assay for rapid and effective detection of APV (excluding Clade C strains). Optimal primers and a probe targeting the APV P4b gene were designed and screened. This method offers accurate results comparable to PCR while significantly reducing the reaction time, providing a reliable tool for initial APV screening and detection in routine laboratories and field environments.

## Materials and methods

### Multiple-sequence alignment and phylogenetic analysis of avipoxvirus

The *P4b* gene sequences from various avian species, including chicken, pigeon, albatross, penguin, turtledove, canary were retrieved from GenBank (Supplementary Table S1). Sequence similarity was analyzed using BLAST through the NCBI database.^1^ The nucleotide sequences of these avipoxvirus strains were compared using MegAlign software. Phylogenetic trees based on the full-length P4b genes were constructed using the neighbor-joining method and the Kimura 2-parameter model in MEGA 6.0. Bootstrap analysis was performed with 1,000 replicates to assess the robustness of the tree (24).

### Design and synthesis of MIRA primers and probe

Full length *P4b* gene sequences of avipoxvirus from various isolates were aligned, and a conserved region was selected as the target for primer and probe design. Following the guidelines provided by AMP-Future Biotech Company (Changzhou, China), MIRA primers targeting the P4b gene were designed. A fluorescent probe complementary to the target region between the upstream and downstream primers was also designed. The primers were approximately 30 nucleotides in length, and the expected amplicon size ranged from 150 to 300 base pairs. The probe was labeled with a FAM fluorescent marker at the 5′-end, a tetrahydrofuran (THF) moiety in the center, and a C3 spacer at the 3′-end. A total of three upstream primers and three downstream primers were designed to pair with the probe, resulting in nine primer pairs in total. The MIRA primers and probe for avipoxvirus detection are listed in Table 1. All sequences were synthesized by Sangon Biotech (Shanghai, China) and purified by high-performance liquid chromatography (HPLC).

**Table 1:** The primers and probes used in this study.

Prime	Sequence (5′-3′)	Source
APV-F1:	CTATATTAGTTGCTTTATTCGGCGTTAAGCTAC	This study
APV-F2:	CTATGGTATTAGGTGATAGTTATAGTCTTATG	
APV-F3:	GTTATAGTCTTATGAAACAATTGTATAACTCG	
APV-R1:	CTGGCTCTATGTATTTCACTAGATACAGAATC	
APV-R2:	CTCAGCATAGTATGAATACTGGCTCTATGTA	
APV-R3:	CACAATCCTTGCTGTAGAATATACCTAATCTC	
APV -Probe:	ATATGTTGTTGATTAATAGGCTTACTGAAGA[FAM]T[THF][BHQ]CCTATTATCTTTACG-[3’C3spacer]	
qAPV-probe	FAM-ATCTCCGCCGTCGCAACTTCCA-BHQ1	Hauck et al. (14)
qAPV-F	TCAGCAGTTTGTTACAAGACA	
qAPV-R	CCATTTCCGTGAATAGAATAGTAT	
FWPV-F	CGGTAGCTTAACGCCGAATA	Lee (13)
FWPV-R	CAGCAGGTGCTAAACAACAA	

### Virus strains and plasmids

The fowlpox virus strains QZ01 was isolated from the diseased chickens exhibiting fowlpox symptoms in Zhejiang province. Various other avian disease viruses, including Newcastle disease virus (NDV), Avian infuenza virus (AIV H9), Infection lnyngotrcheitis virus (ILTV), Infectious bursal disease virus (IBDV), Avian leukosis virus (ALV), Fowl adenovirus serotype 4 (FAdV-4) and Influenza B Virus (IBV) strains, Herpesvirus of turkeys (HVT) were maintained in our laboratory. Viral nucleic acids were extracted using a kit (Jifan, Changzhou, China) following the manufacturer’s instructions. The extracted DNA was stored at −20°C. Subsequently, the full length of virion core protein (*P4b*) gene of FPV was amplified by PCR (TOYOBO, Shanghai, China). PCR fragments were purified from agarose gels and cloned into the pMD-19 T plasmid using Takara kit (Takara, Beijing, China), generating the recombinant plasmid pMD-P4b. The concentration of recombinant Plasmid pMD-P4b was then purified and quantified using an ND-2000c spectrophotometer (NanoDrop, Wilmington, USA). To calculate the copy number of pMD-P4b, the following formula was used: number of plasmids in copies/μL = (6.02 × 10^23^) × (plasmid concentration in ng/μL)/(genome length in bp) × (10^9^) × 660. The plasmid was diluted achieve a copy number of 5 × 10^5^/μL. Serial 10-fold dilutions ranging from 5 × 10^5^ to 5 copies/μL were prepared for use in subsequent assays.

### Multi-enzyme isothermal rapid amplification reaction

The MIRA was performed using the DNA isothermal rapid amplification kit (#WLE8202KIT, AMP-Future Biotech, Changzhou, China), according to the manufacturer’s instructions. Different primers were designed and diversity combinations were utilized to screen for the most suitable primer pairs. For the MIRA assay, a 50 μL reaction mixture comprised 29.4 μL of buffer A, 2.5 μL of buffer B, 2 μL of each forward/reverse primer (10 μM), 0.6 μL of probe, 2 μL DNA template and remaining volume filled with ddH_2_O. The reaction tube was vortexed shortly ensure thorough mixing of the reagents and centrifuged briefly. Then the reaction mixture was immediately placed in a ABI QuantStudio 5 Real-Time Cycle (Thermal, USA) and incubated at 40°C for 20 min. The fluorescence signal was monitored, and the amplification products were applied to the visual analysis under a blue light imager. The generated products were purified with phenol followed by centrifugation at 5,000 rpm for 5 min and separated on a 2% agarose gel through electrophoresis. After electrophoresis, the products were visualized using an automatic digital gel image analysis system (BioRad, California, USA).

### Optimum temperature for the MIRA assay

To determine the optimal temperature for MIRA amplification system, a gradient reaction temperature optimization were conducted. Different dilution of FPV nucleic acid (10 and 1,000 times dilution) was prepared as test templates. The reaction temperatures tested ranged from 25 to 50°C, with intervals at 5°C (25, 30, 35, 40, 45, and 50°C). By systematically varying the temperature, the aim was to identify the temperature condition that provided the highest amplification efficiency for FPV detection.

### Sensitivity and specificity analysis of the MIRA assay

To evaluate sensitivity of the MIRA assay for FPV, the standard plasmid pMD-P4b, previously constructed, was used. The plasmid DNA was diluted in a 10-fold gradient from 5 × 10^5^ to 5 copies/μL to create a series of test samples. One microliters of each dilution were then amplified using the MIRA assay to determine the detection limit, with RNase-free ddH₂O used as a negative control. The specificity of the MIRA assay was further tested against a panel of other avian viruses, including FPV, PPV, NDV, AIV, ILTV, IBDV, ALV, FAdV-4, IBV, and HVT. Nucleic acid templates were extracted from each virus, and the MIRA assay was conducted to confirm the assay’s ability to accurately detect FPV and PPV without cross-reacting with other pathogens.

### Optimization of reaction time for the MIRA assay

To determine the optimal reaction time for the MIRA assay in detecting FPV, the test was performed at three different time points: 10, 15, and 20 min. DNA templates, based on the results from the sensitivity analysis, were selected, using both high and low copy numbers of nucleic acid to assess the fastest detection time.

### Quantitative PCR analysis

The FPV *P4b* based qPCR assay was performed using the AceQ Universal U & Probe Master Mix (Vazyme, Nanjing, China). The primes and probe was used according to the reported assay (14). The conditions for the qPCR assay was performed in a 20 μL The qPCR assay reaction mixture contained 10 μL of 2 × AceQ Universal U + Probe Master Mix, 0.4 μL of 10 μM of each primers, TaqMan Probe 0.2 μL of 10 μM, 8 μL of ddH_2_O and 1 μL of DNA template. The reaction conditions were 37°C for 2 min, 95°C for 5 min, followed by 40 cycles of 95°C for 10 s and 60°C for 30 s.

### Evaluation of clinical sample performance

A total of 86 clinical samples including throat swab and tissue samples were collected from chicken (75 samples) and pigeon (11 samples) (Supplementary Table S2). The collected tissue samples were homogenized in phosphate-buffered saline (PBS), used for nucleic acid extraction. The MIRA method developed in this study was employed for detection. The outcomes were compared with those of qPCR to determine the positive rate. By comparing the results obtained from these two assays, we aimed to evaluate the performance and reliability of the MIRA assay for the detection of FPV in the collected samples.

## Results

### Alignment and phylogenetic analysis of the *P4b* gene

**Figure 1:**
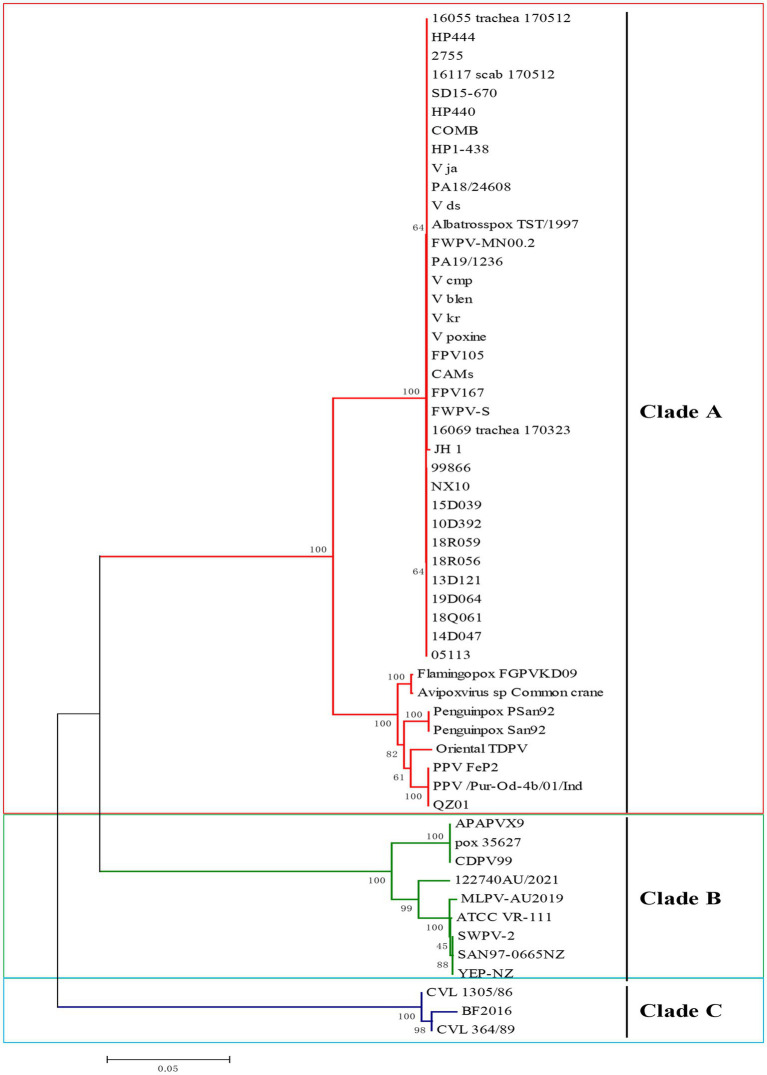
Phylogenetic analysis of *P4b* gene of avipox virus (APV) based on nucleotide sequences. Phylogenetic analysis based on the *P4b* gene from clinical isolates in this study and representative strains of APV available from the GenBank database. APV is classified into 3 clades, A, B, and C. The isolated FPV and PPV strains in the current study belonged to clade A. The tree was constructed with MEGA 7.0 software using the neighbor-joining method with 1,000 bootstrap replicates.

The field strains of FPV JH-1 (GenBank ID: PQ816339) and PPV QZ01 (GenBank ID: PQ816340) were isolated from diseased chicken and pigeons using chicken embryo, respectively. The *P4b* genes were then amplified and cloned into pMD-T19 plasmids. The full-length *P4b* gene sequences were determined and deposited in GenBank. Additional nucleotide sequences of APVs from various avian species, retrieved from NCBI (Supplementary Table S1), were included in the alignment. The phylogenetic analysis revealed three main clades in the tree: Clade A, which includes fowlpox viruses, pigeonpox viruses, penguinpox viruses, Albatrosspox and Oriental TDPV; Clade B, which consists of canarypox virus, albatrosspox virus, penguinpox virus 2 and finch poxvirus; and Clade C, which includes psittacine pox-like viruses (Figure 1).

The multiple sequence alignment analysis of P4b gene in this study revealed that the sequence of FPV JH-1 showed 100% similarity identity with other reported FPV virus such as V_ds, HP1-438, V105, HP444 and CAMs. And all the FPV had over 99% similarity with each other. Indeed, the PPV field strain in this study showed an average of 100% nucleic acids identity to the other PPV FeP2 and Pur-Od-4b/01.All the Clade A group viruses displayed high similarity between 92.6 and 100%, respectively. The Clade B group viruses demonstrated similarity between 76.8 and 78.4% with Clade A viruses. Clade C isolates distinct far from 73.9–76.1% with Clade A (Supplementary Figure S1). Thus, the high similarity of sequence of Clade A strains provide a possible for design one pairs to diagnosed the avipoxvirus mainly for FPV, PPV.

### Primer screening and identification

**Figure 2:**
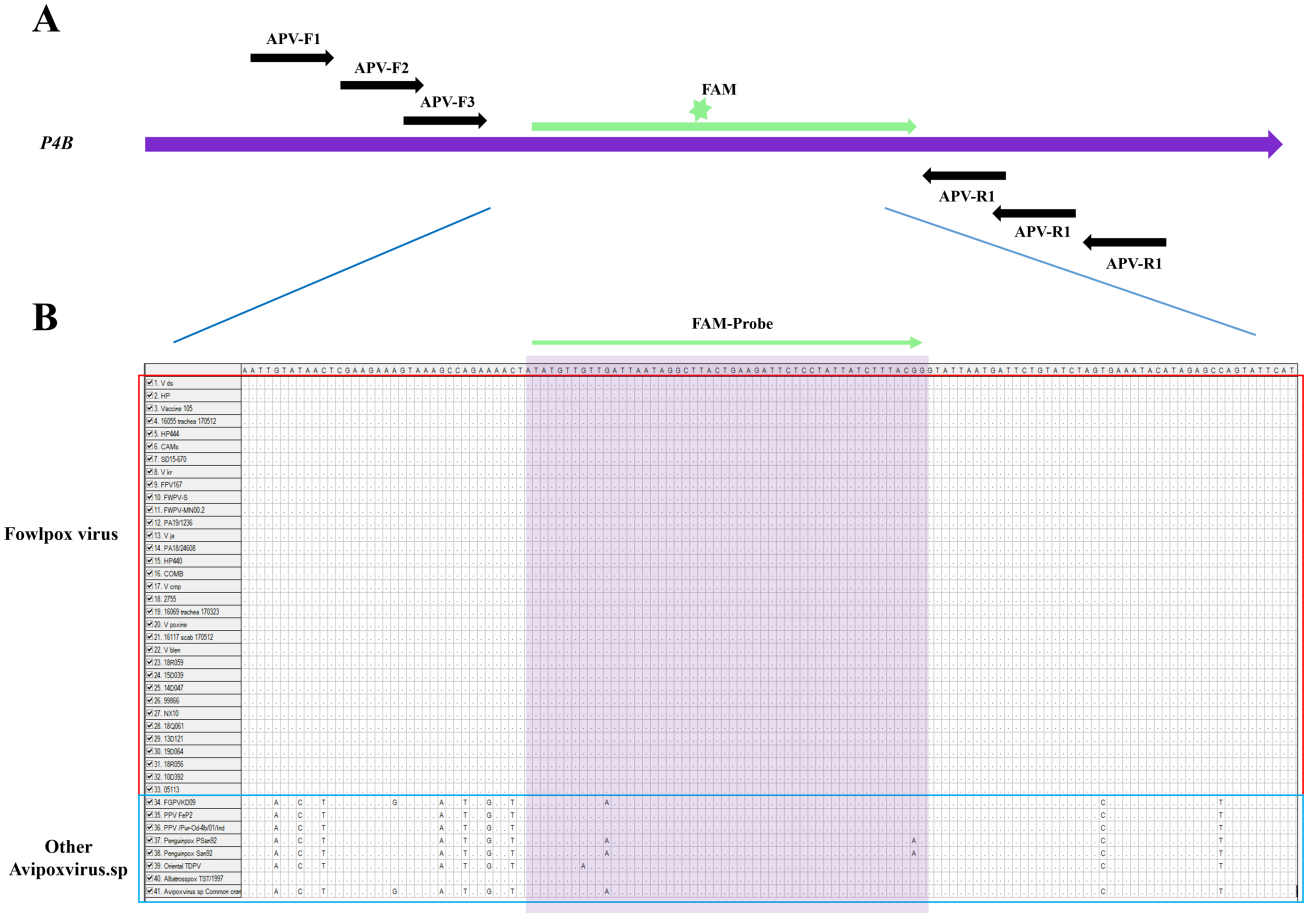
Schematic diagram of the primer design for MIRA. **(A)** The primes targeting to the conserved *P4b* region of Clade A APV. Three forward and three reverse candidate primers that surround the probe are indicated with arrows of different lengths. Forward primers, APV-F1, APV-F2, APV-F3, Forward primers, Reverse primers, APV-R1, APV-R2, APV-R3. The probe with FAM in the middle region and C-C3Space. **(B)** The information of Clade A APV were list, the name of isolates of the sequences are indicated on the left. Nucleotide residues that match the majority are indicated by dots. The conserved sequence for probe primers design were selected.

In this study, an ideal exo probe was designed within the conservative region of *P4b* gene to target both Clade A avipoxviruses. The positions of the primers and probe within the sequences are shown in Figure 2 and the probe located sequences displayed 100% identified in both FPV and PPV. The design incorporated a FAM fluorescent marker in the middle of the probe, with the 3′ end modified with a C3 spacer. Three upstream and three downstream candidate primers were selected to match the probe region. The MIRA primers were then screened for efficient amplification, following the manufacturer’s instructions for the reaction.

**Figure 3:**
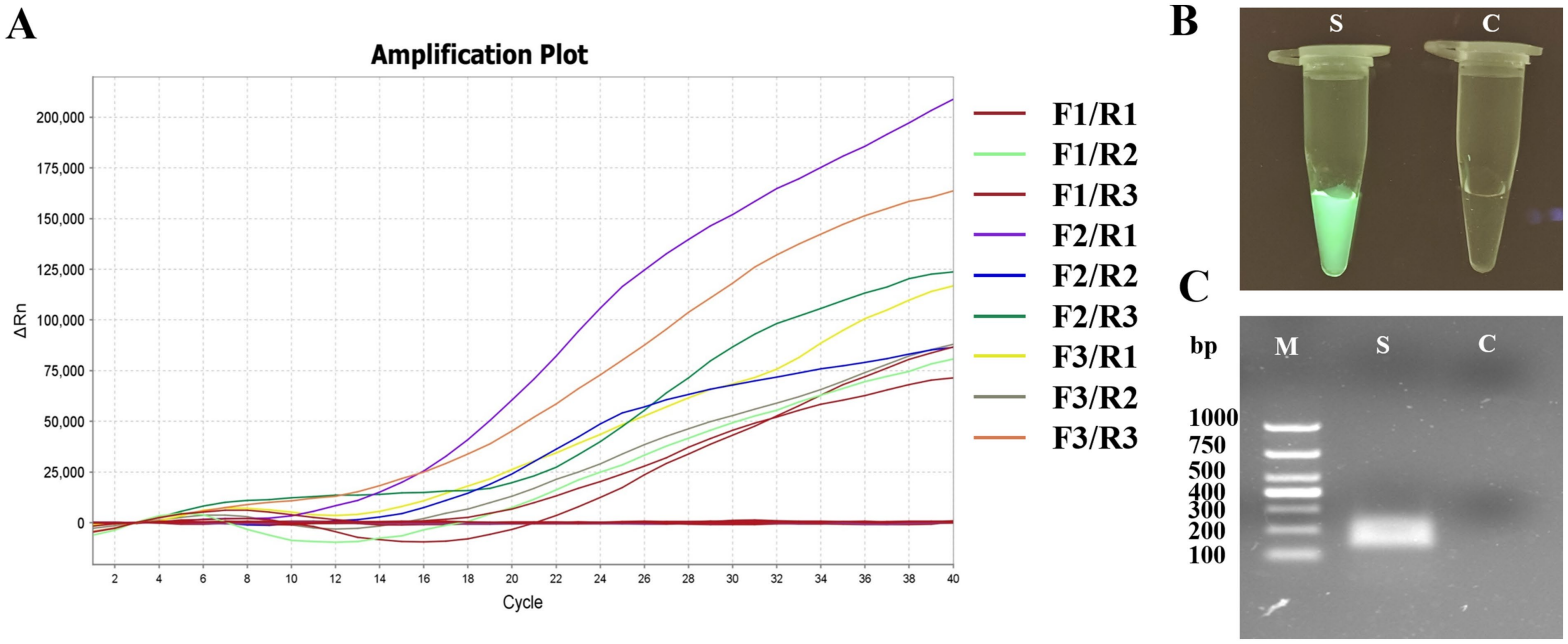
Primer screening tests of MIRA for APV detection. **(A)** The results of real-time MIRA by screening all nine pairs primers with the fluorescence signal. **(B)** The reaction tube with F2/R1 MIRA primers amplification and observed under Blue light. S: positive, C: negative control. **(C)** Agarose gel electrophoresis of F2/R1 MIRA primers amplification products. M, 1,000 DNA marker; S, positive with 163 bp.

A total of nine pairs of candidate forward and reverse primers were evaluated using the MIRA assay, with diluted FPV strain JH-1 DNA as the template. Fluorescence signal intensity was measured, and the results showed that the signal produced by the F2/R1 primer pair was the brightest (Figure 3A). Moreover, the amplification products were visible under blue light with green fluorescence (Figure 3B), and 2% agarose gel electrophoresis confirmed that this primer pair produced a distinct and specific band of 163 bp (Figure 3C). As a result, the F2/R1 primer pair, along with its corresponding probe, was selected for further studies in the MIRA assay.

### Optimization of the reaction temperature

**Figure 4:**
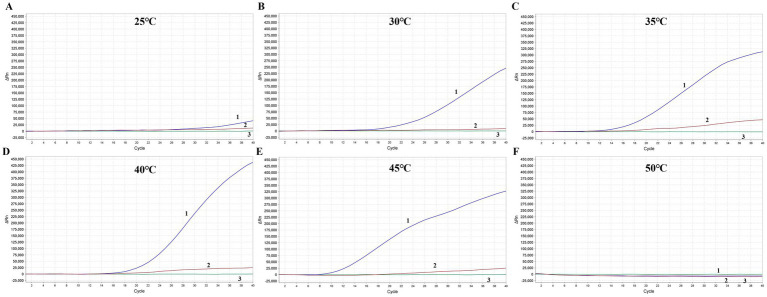
Optimization of the reaction temperature for MIRA assay. The optimal amplification reaction time was determined by examining various temperature settings ranging from 25°C **(A)**, 30°C **(B)**, 35°C **(C)**, 40°C **(D)**, 45°C **(E)** and 50°C **(F)**. APV nucleic acid with two different dilution ration were applied. 1: Blue line, APV nucleic acid with 10 times dilution. 2: Red line, APV nucleic acid with 1,000 times dilution. 3: Green line, Negative control.

To determine the optimal amplification temperature for the MIRA assay, the test was conducted following the manufacturer’s instructions. Two different FPV DNA templates were used in the experiment. The basic MIRA assay was performed at six different temperatures ranging from 25 to 50°C (25, 30, 35, 40, 45, and 50°C) for 20 min. Fluorescence signals were used to detect the target amplicon. The results showed that the best performance occurred between 35 and 45°C, with significant fluorescence observed (Figure 4). The strongest fluorescence signal was observed at 40°C, which was selected as the optimal reaction temperature for subsequent experiments. In contrast, the assays conducted at the lower temperature of 25°C and the higher temperature of 50°C failed to detect FPV.

### Specificity determination of MIRA

**Figure 5:**
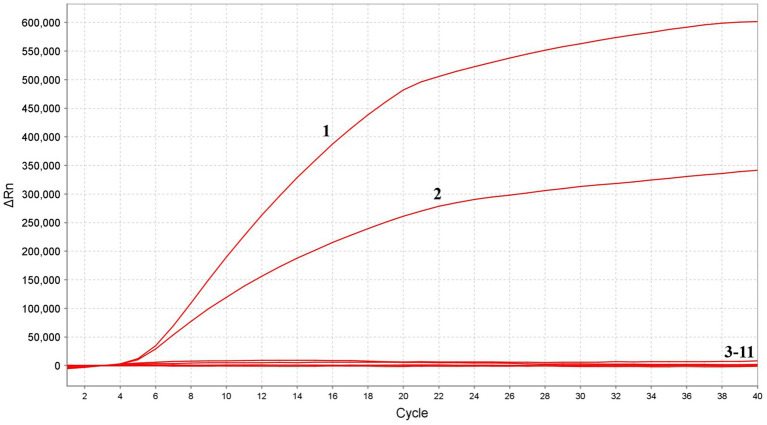
Specificity of MIRA assay. The positive sample 1(FPV) and sample 2(PPV) produced amplification fluorescence signals, whereas other avian viruses produced no amplification signals. 3–11: NDV, AIV, ILTV, IBDV, ALV, FAdV-4, IBV, HVT and ddH_2_O.

In the analytical specificity analysis, the MIRA assay was performed using the nucleic acids extracted from various viruses, including FPV, PPV, NDV, AIV, ILTV, IBDV, ALV, FAdV-4, IBV and HVT as templates, ddH_2_O was the negative control. The results of the specificity assay revealed that when the FPV and PPV nucleic acid used as template, significant fluorescence signals could be detected, and there was no cross-reaction with other pathogens, which indicated that the established method had high specificity (Figure 5).

### Analytical sensitivity of the MIRA

**Figure 6:**
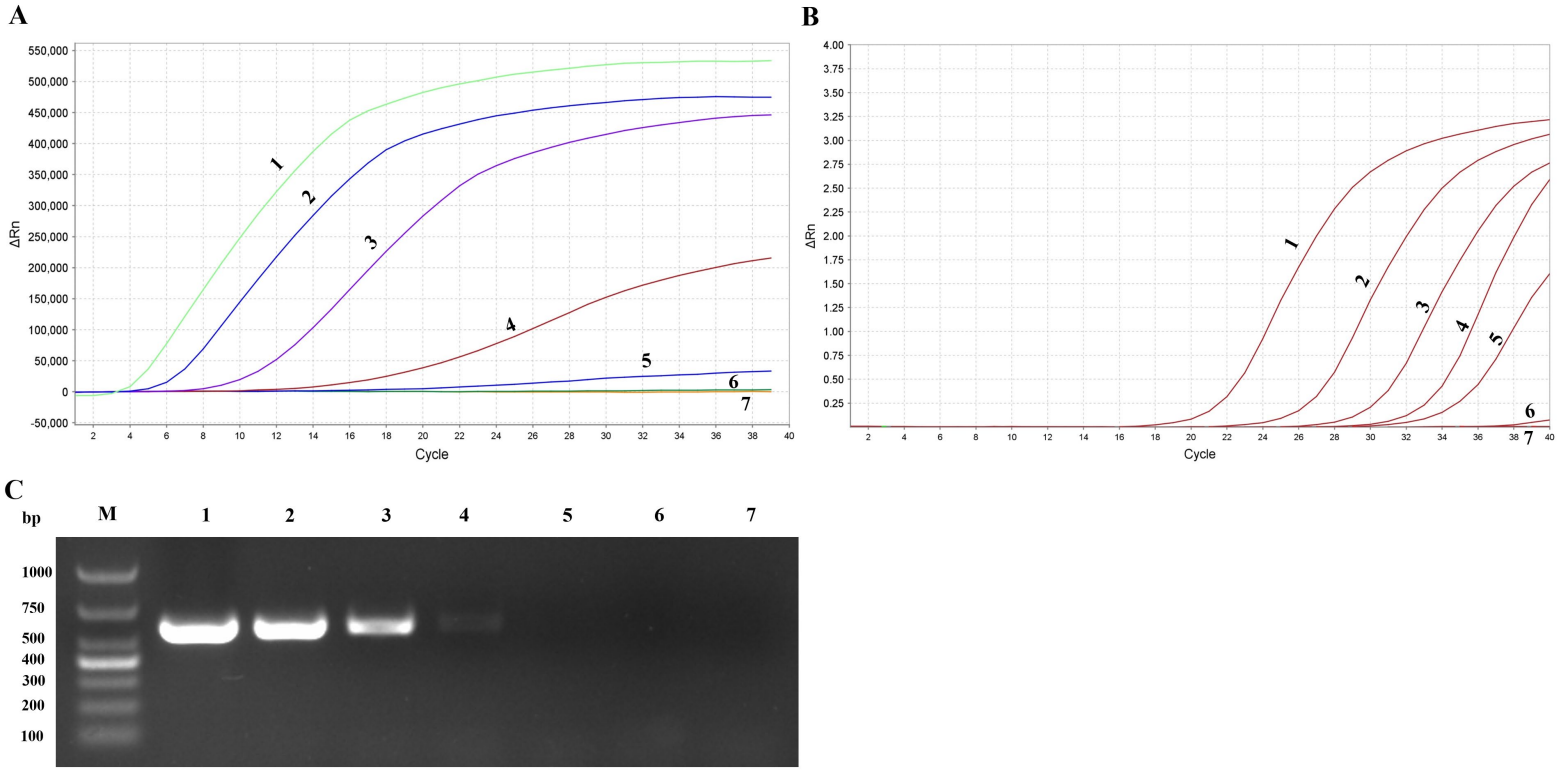
Sensitivity of MIRA assay, qPCR and PCR assay. Serially diluted APV positive plasmid of targeted gene (1–7: 5 × 10^5^copies/μL-5 copies/μL and ddH_2_O) were tested by MIRA **(A)**, qPCR **(B)**, and PCR **(C)** detection assay.

The standard recombinant positive plasmids of FPV and PPV were diluted to various concentration ranging from 5 × 10^5^ to 5 copies/μL and detected using the MIRA and PCR systems. The detection limit of MIRA was evaluate and compared the results with those obtained from the PCR and qPCR assay. The results revealed that the developed MIRA assay displayed a detection limit of 50 copies for both FPV and PPV as indicated by a weak fluorescence value, while the higher concentration produce significant signals (Figure 6A). However, the sample 5 copies/μl and the negative control (ddH_2_O) did not exhibit any signal. Additionally, it was detected in qPCR assay with template 50 copies/μl, but it still cannot detect the 5 copies/μl (Ct > 37) template (Figure 6B). In the ordinary PCR test, the detection limit with 500 copies/μl (Figure 6C), which showed the sensitivity of conventional PCR is not as good as that of MIRA and qPCR. In summary, the sensitivity detection achieved by the MIRA assay was comparable to that of the qPCR assay. This suggests that the established MIRA assay is suitable for the detection of FPV and PPV and can provide reliable results.

### Optimization of the reaction time

**Figure 7:**
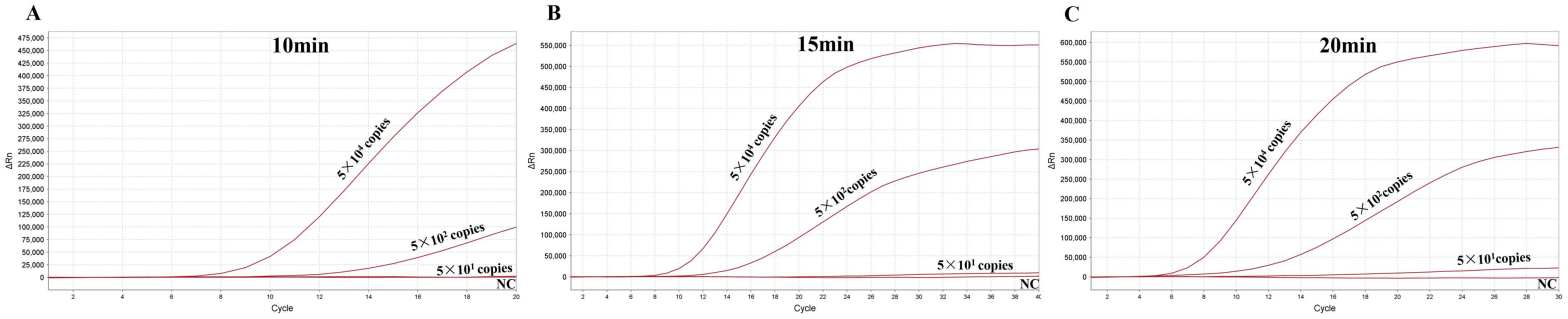
Optimization of the reaction time for MIRA. The optimal amplification time was determined by examining different time: 10 **(A)**, 15 **(B)**, and 20 **(C)** min.

To evaluate the shortest time for avipoxvirus detection with the MIRA assay, various reaction times (10 min, 15 and 20 min) were evaluated to determine the minimal amplification time required. Three different template concentrations were used in the experiments. The results indicated that the MIRA assay could complete detection of APV in just 15 min, even with the lowest template copies, which was similar to the results obtained at 20 min (Figure 7). Additionally, the MIRA assay successfully detected high-copy templates within 10 min. These findings demonstrate that the MIRA amplification method offers a rapid and efficient approach for detecting APV.

### Evaluation of the MIRA with clinical samples

To further evaluate the clinical performance of the APV MIRA assay, 86 clinical samples, including tissue and swab samples, were tested for validation. Nucleic acid extraction from the samples was performed using both the MIRA assay and qPCR. The detection results from both methods were then compared. Of the 86 clinical samples, all 35 positive samples and 51 negative samples were accurately identified by both methods, most of the positive samples belong to FPV (34/75), only 1 sample belong to PPV (1/11), as shown in Table 2. Notably, the results demonstrated that both the MIRA assay and qPCR provided consistent detection rates. However, the MIRA assay offered the advantage of faster results compared to qPCR, underscoring its potential as a rapid and reliable diagnostic tool for APV in clinical samples. The successful validation of the MIRA assay indicates its suitability for on-site testing, requiring minimal laboratory equipment and professional expertise. This enhances the convenience and accessibility of APV detection, especially in environments where timely diagnosis is essential for effective disease management and control.

**Table 2:** Comparison of qPCR and MIRA results for clinical samples.

Results	MIRA positive	MIRA negative	Coincidence rate	Kappa (κ)
qPCR positive	35	0	100%	1
qPCR negative	0	51		
Total	35	51		

## Discussion

The most well-known poxvirus is the Monkeypox virus (MPXV), a zoonotic disease that has become a growing global health concern due to its epidemic potential and rapidly increasing incidence (25). Historically, smallpox was one of the most notorious poxviruses, causing red spots and pustules on the skin, often leading to death in humans (26). Smallpox dates back to ancient times and was once considered a curse of nature. Avipoxvirus (APV), another ancient viral pathogen, causes infectious diseases in both domestic and wild bird species of all ages, genders, and breeds (27). APV was one of the earliest diseases described in poultry, with detailed studies beginning in the 1870s through microscopic observations of cytoplasmic eosinophilic inclusion bodies. APV presents clinically similar symptoms to other avian diseases, with characteristic pox lesions on the skin. It has been effectively controlled through vaccination and strict hygiene measures, which it once as sever disease agent in poultry with highly mortality. However, APV has reemerged in recent years, with several outbreaks reported in both poultry and pigeons, causing significant economic losses. There have been reports of outbreaks occurring even in vaccinated flocks, raising questions about whether this is due to inadequate vaccination coverage or the emergence of novel viral variants.

Avian poxvirus causes three main forms of disease and he cutaneous form is characterized by wart-like lesions on the comb, wattle, legs, feet, and other unfeathered parts of the body (27). These lesions, which can vary in color from yellow to brown, are indicative of epithelial hyperplasia and are the most common clinical manifestation of APV infection. This form of the disease can lead to significant mortality. The diphtheritic form involves proliferative necrosis of the mucous membranes in the respiratory and/or digestive tracts, often leading to severe complications. The ocular variant affects the eyelids, causing epithelial hyperplasia that frequently results in ulcerated growths in the feather follicles. High mortality is common in the diphtheritic form due to the accumulation of yellowish, cheesy material in the mucous membranes of the mouth, nose, and larynx. Avian can be infected by one or more forms of the disease, sometimes accompanied by conjunctivitis. New challenges have emerged in managing APV, including increasing outbreaks on previously vaccinated farms, reports of atypical or more severe disease presentations, and a lack of understanding regarding the interaction between APV and the integrated retrovirus (REV) provirus, which may represent a pathotypic variant in the field (28, 29). REV, a member of the Gammaretrovirus genus in the Orthoretrovirinae subfamily of Retroviridae, is associated with diseases in chickens, ducks, geese, pigeons, and other avian species (30, 31). REV integration into the APV genome may occur during co-infections of a host with both FPV and REV (32). While the integration of REV into pigeonpox virus has not been reported, there is anecdotal evidence suggesting that field strains of FPV containing REV may be more problematic, potentially due to increased virulence, resistance to vaccine-induced immunity, or enhanced virus fitness (8). Therefore, the effective detection of APV is critical for disease control and management.

The *P4b* core protein gene is a distinctive genetic marker for poxviruses (33). Its highly conserved nature enables the molecular diagnosis and differentiation of poxviruses across different host species, as well as among various strains within the same species. The PCR diagnosis methods that target the amplification of the *P4b* gene are highly effective for detecting APV infections. Phylogenetic analysis of the *P4b* gene reveals that FPV and PPV fall under Clade A, while isolates in Clade B and C are distinct from the A group (34). In the avian industry, the chicken and pigeon are the most important compared to other wild bird like albatross, oriental turtle dove and penguin. In this way, a MIRA assay was developed for the detection of Clade A APV, specifically targeting FPV and PPV strains in this study.

**Figure 8:**
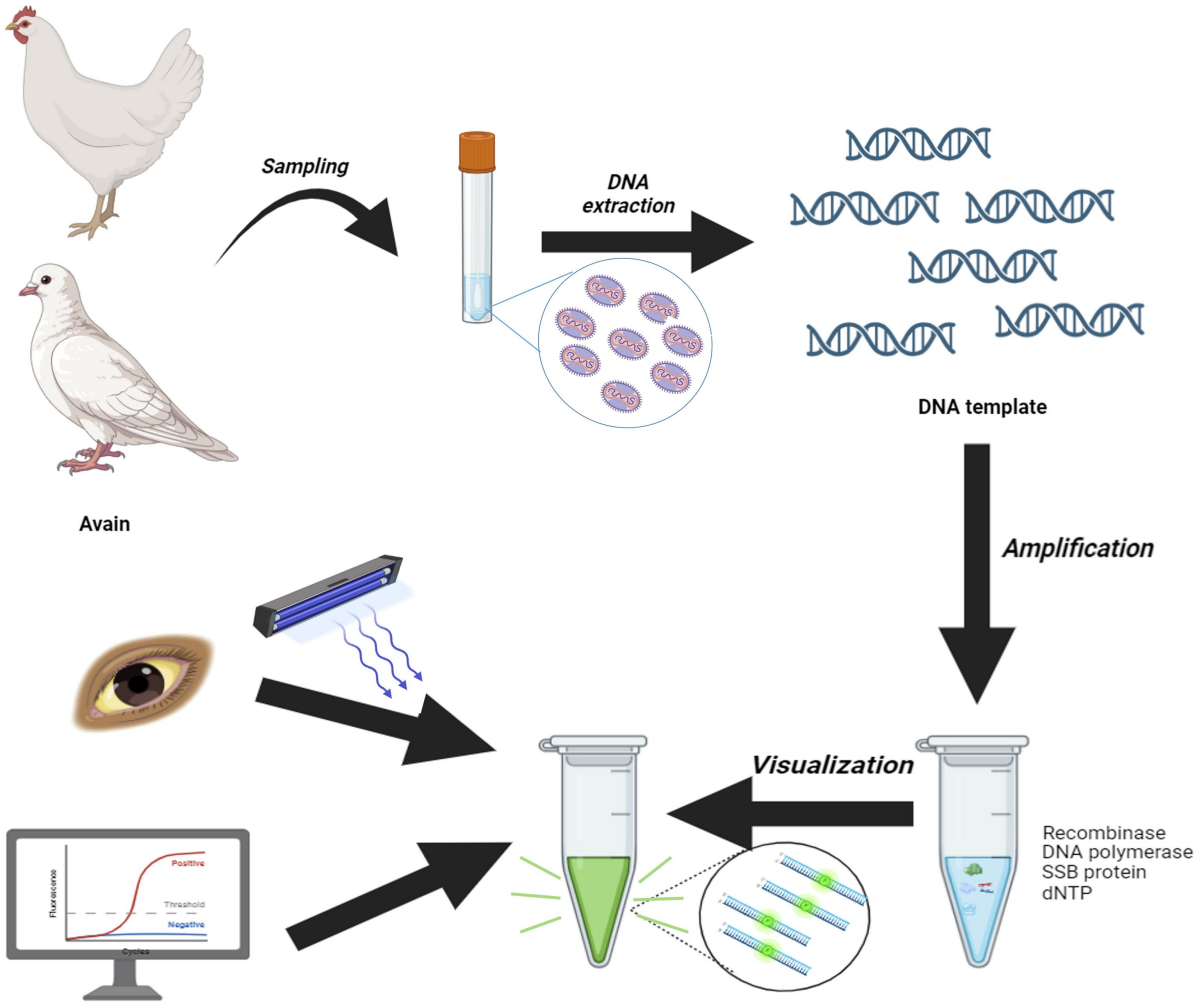
Scheme summarizing the entire process.

MIRA offers a promising alternative for point-of-care testing (POCT), overcoming many of the limitations associated with traditional PCR methods, which typically require specialized equipment and at least 1 h for completion. In contrast, MIRA provides intuitive results that can be directly observed with the naked eye under blue light. While PCR often requires agarose gel electrophoresis and gel imaging systems to visualize amplicons, qPCR enables continuous monitoring through fluorescence signal accumulation by professional machine. MIRA technology utilizes a combination of four functional proteins DNA helicase, recombinase RecA, single-stranded binding (SSB) protein, and DNA polymerase, which work together to facilitate rapid D-loop formation, completing the reaction within 20 min at a constant temperature. Compared with other isothermal amplification including RPA and LAMP, while RPA relies on T4 UvsX and *Escherichia coli* UvsX recombinases, MIRA employs the *Streptomyces azureus* RecA (SC-recA) recombinase system. This enzymatic optimization confers enhanced reaction stability across diverse environmental conditions compared to conventional RPA. Furthermore, MIRA demonstrates significant practical advantages over LAMP technology. The latter requires six pairs of primers for target recognition, whereas MIRA achieves comparable amplification efficiency with only a single primer pair. This streamlined design substantially simplifies assay development and broadens its applicability in resource-limited settings. Previous studies on target gene detection with MIRA have identified minor variations in the optimal temperature, with 40°C typically yielding the best results. In this assay, we ascertained that temperatures of 35°C and 45°C also displayed high reaction activity, comparable to that at 40°C. Therefore, this study introduced a new rapid amplification approach for APV detection using MIRA, significantly reducing the detection time to as little as 15 min and simplifying the testing process (Figure 8). The MIRA assay developed here is highly effective for amplifying small amounts of nucleic acid, with a minimum detection limit of 50 copies/μL. Furthermore, the MIRA detection method demonstrates exceptional specificity, achieving 100% accuracy compared to qPCR. Importantly, it does not cross-react with other common viruses in avian species, such as NDV, AIV, ILTV, IBDV, NDV, ALV, FAdV-4, IBV and HVT, ensuring reliable identification of APV and minimizing the risk of false-positive results and facilitating accurate diagnosis. The rapid turnaround time is essential for timely intervention, facilitating quick response measures to control outbreaks and protect avian populations.

Since the initial identification of APV, various PCR-based assays including PCR, SYBR Green-based qPCR and TaqMan probe-based qPCR have been developed. However, these methods often face challenges in meeting the requirements of grassroots detection and operational convenience, typically requiring substantial time for diagnosis. The ordinary PCR method with 578 bp products was wildly applied in FPV detection since 1997. It has also been verified both for FPV, PPV, TDPV. Here the results have been demonstrated the MIRA showed better sensitivity than ordinary PCR. In contrast to qPCR, MIRA assays do not require costly instruments, and compared to other isothermal amplification methods like LAMP, MIRA assays are simpler to design. Furthermore, MIRA assays are faster than both qPCR and LAMP, making them better suited for rapid, on-site detection.

The development and application of rapid pathogen detection methods are crucial for the prevention and control of clinical infectious diseases. The MIRA technology has been successfully utilized to detect specific pathogens in both humans and animals, such as pseudorabies virus (19), classical swine fever virus (35), African swine fever virus (36), monkey poxvirus (37), SARS-CoV-2, and hepatitis C virus (18, 38). To our knowledge, this is the first use of the MIRA assay for detecting APV. This study not only demonstrates the effectiveness of MIRA in detecting APV but also lays the groundwork for its future use in detecting other animal pathogens. This breakthrough has the potential to transform pathogen detection in veterinary medicine by offering rapid and sensitive diagnostic solutions.

## Conclusion

In summary, MIRA represents a major advancement in pathogen detection, providing several advantages over traditional methods. Its rapid turnaround, increased effectiveness, and superior sensitivity make it especially suitable for field applications, where prompt detection is essential for effective disease management. The MIRA assay developed in this study can accomplish detection within 15 min, demonstrating excellent specificity and sensitivity. These benefits make it a valuable tool for controlling and investigating APV infections.

## Data Availability

The datasets presented in this study can be found in online repositories. The names of the repository/repositories and accession number(s) can be found in the article/Supplementary material.

## Footnotes

^1^ https://blast.ncbi.nlm.nih.gov/Blast.cgi
