# Supplementary material for: Rapid detection of avipoxvirus using a fluorescent probe-based multienzyme isothermal amplification assay
Source: Front Vet Sci. 2025 May 14;12:1601685. doi: 10.3389/fvets.2025.1601685 (PMC12116516; doi:10.3389/fvets.2025.1601685)
Supplement: Supplementary file 1 [file Table_1.docx]

**Table S1 The information list of avipoxvirus *P4b* analyzed in this study.**

| **Name** | **Type** | **Clade** | **GenBank no.** |
| --- | --- | --- | --- |
| V_ds | Fowlpox virus | A | MW558079.1 |
| HP444 | Fowlpox virus | A | M25781.1 |
| Fpv105 | Fowlpox virus | A | OR800602.1 |
| 16055_trachea_170512 | Fowlpox virus | A | MF766430.1 |
| FPV-CAMs | Fowlpox virus | A | OK558608.1 |
| SD15-670 | Fowlpox virus | A | MH719203.1 |
| V_kr | Fowlpox virus | A | MW558080.1 |
| SarPox | Fowlpox virus | A | KU551306.1 |
| HP 1-438 | Fowlpox virus | A | AJ581527.1 |
| FWPV-S | Fowlpox virus | A | MW142017.1 |
| FWPV-MN00.2 | Fowlpox virus | A | MH709124.1 |
| PA19/1236 | Fowlpox virus | A | OK345040.1 |
| V_ja | Fowlpox virus | A | MW558081.1 |
| PA18/24608 | Fowlpox virus | A | OK345039.1 |
| COMB | Fowlpox virus | A | OK558609.1 |
| V_cmp | Fowlpox virus | A | MW558078.1 |
| 2755 | Fowlpox virus | A | MW558073.1 |
| 16069_trachea_170323 | Fowlpox virus | A | MF766431.1 |
| V_poxine | Fowlpox virus | A | MW558076.1 |
| 16117_scab_170512 | Fowlpox virus | A | MF766432.1 |
| V_blen | Fowlpox virus | A | MW558077.1 |
| 18R059 | Fowlpox virus | A | MW558071.1 |
| 15D039 | Fowlpox virus | A | MW558068.1 |
| 14D047 | Fowlpox virus | A | MW558067.1 |
| 99866 | Fowlpox virus | A | MW558075.1 |
| NX10 | Fowlpox virus | A | KX196452.1 |
| 18Q061 | Fowlpox virus | A | MW558069.1 |
| 13D121 | Fowlpox virus | A | MW558072.1 |
| 19D064 | Fowlpox virus | A | MW558072.1 |
| 18R056 | Fowlpox virus | A | MW558070.1 |
| 10D392 | Fowlpox virus | A | MW558065.1 |
| 05113 | Fowlpox virus | A | MW558074.1 |
| FGPVKD09 | Flamingopox virus | A | NC_036582.1 |
| FeP2 | Pigeonpox virus | A | KJ801920.1 |
| PPV /Pur-Od-4b/01/Ind | Pigeonpox virus | A | ON375849.1 |
| PSan92 | Penguinpox virus | A | KJ859677.1 |
| San92 | Penguinpox virus | A | FJ948105.1 |
| TDPV | Oriental turtle dovepox virus | A | OQ547902.1 |
| TST/1997 | Albatrosspox virus | A | OK348853.1 |
| crane-1 | Avipoxvirus sp | A | PP341421.1 |
| ATCC VR-111 | Canarypox virus | B | AY318871.1 |
| SAN97-0665NZ | Albatrosspox virus | B | MW365933.1 |
| YEP-NZ | Penguinpox virus 2 | B | MW296038.1 |
| SWPV-2 | Shearwaterpox virus | B | KX857215.1 |
| MLPV-AU2019 | Mudlarkpox virus | B | MT978051.1 |
| 122740AU/2021 | Crowpox virus | B | ON408417.1 |
| APAPVX9 | Apapanepox virus | B | OQ865377.1 |
| pox_35627 | Finch poxvirus | B | OM869483.1 |
| CDPV99 | Condorpox virus | B | OQ865376.1 |
| CVL 1305/86 | Psittaciformes sp | C | AM050382.1 |
| CVL 364/89 | Psittaciformes sp | C | AM050383.1 |
| BF2016 | Psittaciformes sp | C | MG601780.1 |

**Table S2. Information of samples**

| **Species** | **Farms** | **Provinces** | **Sample** | | **No. of samples** | **APV/PPV positive rate** |
| --- | --- | --- | --- | --- | --- | --- |
| Lohmann | 1 | Zhejiang | throat swabs | | 6 | 0（0%） |
| Hy-Line | 1 | Zhejiang | throat swabs/skin | | 25 | 17（68%） |
| Hy-Line | 1 | Zhejiang | skin | | 12 | 0（0%） |
| Lohmann | 1 | Zhejiang | throat swabs | | 12 | 9（75%） |
| Hy-Line | 1 | Zhejiang | skin | | 7 | 2 (28%) |
| Jingfen Pink | 1 | Zhejiang | skin | | 10 | 5 (50%) |
| Jingfen Pink | 1 | Zhejiang | throat swabs | | 3 | 1（33%） |
| pigeon | 1 | Zhejiang | throat swabs/ skin | | 11 | 1 (9%) |
| **Total** | 8 |  |  | 86 | | 35（41%） |


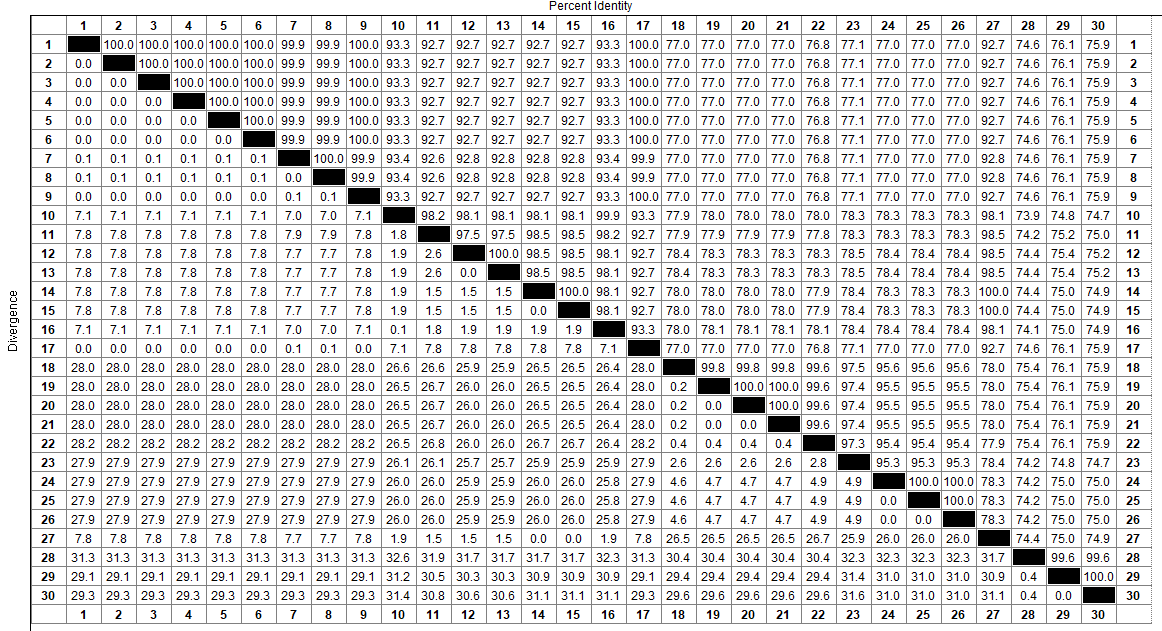


**Figure.S1 The identity of different avipoxvirus.** 1, FPV V_ds; 2, FPV HP1-438; 3, FPV V105; 4, FPV 16055_trachea_170512; 5, FPV HP444; 6, FPV CAMs; 7, FPV 18R059; 8, FPV 15D039; 9, Albatrosspox TST/1997; 10, Avipoxvirus Common crane; 11, Oriental TDPV; 12, Penguinpox San92; 13, Penguinpox PSan92; 14, PPV Pur-Od-4b/01; 15, PPV FeP2; 16, Flamingopox FGPVKD09; 17, FPV JH-1; 18, Canarypox virus ATCC VR-111; 19, Albatrosspox virus SAN97-0665NZ; 20, Penguinpox virus 2 YEP-NZ; 21, Shearwaterpox virus SWPV-2; 22, Mudlarkpox virus MLPV-AU2019; 23, 122740AU/2021; 24, Apapanepox virus APAPVX9; 25, Finch poxvirus pox_35627; 26, CDPV99 Condorpox virus; 27, PPV QZ01; 28, Psittaciformes sp CVL 1305/86; 29, Psittaciformes sp CVL 364/89; 30, Psittaciformes sp BF2016.
